# Continuous or Transient High Level of Glucose Exposure Differentially Increases Coronary Artery Endothelial Cell Proliferation and Human Colon Cancer Cell Proliferation

**DOI:** 10.22074/cellj.2017.4446

**Published:** 2017-05-17

**Authors:** Yoko Shimoda, Yuko Tagaya, Tsugumichi Saito, Eijiro Yamada, Aya Osaki, Yasuyo Nakajima, Atsushi Ozawa, Tetsurou Satoh, Junichi Okada, Shuichi Okada, Masanobu Yamada

**Affiliations:** Department of Medicine and Molecular Science, Gunma University Graduate School of Medicine, Showa-machi, Maebashi, Gunma, Japan

**Keywords:** Cell Proliferation, Erk, Akt

## Abstract

We studied effect of high glucose levels on coronary artery endothelial cell proliferation and human colon cancer cell proliferation. To examine the long-term effect of glucose exposure on cell growth, cells were cultured for 14 days in the absence or presence of 183 mg/dL D-glucose addition in the culture medium. Short effect of elevated glucose levels was examined by addition of 183 mg/dL D-glucose addition in the culture medium for just one hour per day followed by changing the culture to standard medium (5.5 mM D-glucose) during the next 23-hours period. Cell proliferation was estimated by 2,3-Bis (2-methoxy-4-nitro-5-sulfophenyl)-2H-tetrazolium-5-carbox-anilide (XTT) assay and phosphor-Erk western blot analysis. We found that coronary artery endothelial cell proliferation was significantly increased in the culture medium with the acute one-hour addition of 183 mg/dL D-glucose compared to the absence or chronic presence of 183 mg/dL D-glucose addition in the culture medium. In contrast, colon cancer cell proliferation was significantly increased in the continuous presence of 183 mg/dL D-glucose addition in the culture medium compared to the acute one-hour addition of glucose. The extent of Erk2 phosphorylation paralleled with the relative changes in cellular proliferation in both cell types. Taken together, these results suggested that continuous or transient high level of glucose exposure differentially effects coronary artery endothelial and human colon cancer cell proliferation.

Diabetes mellitus patients have higher risk for both malignant tumor development such as colon cancer and atherosclerosis leading to coronary artery disease (1, 2). High levels of glucose exposure were reported to directly affect cell proliferation. For example, human aortic endothelial cell proliferation chronically exposed to high level of glucose (22.2 mM for 72 hours) was suppressed (3); whereas in cultured colon, cancer cell proliferation (11 mM) was increased under diabetogenic glucose concentration (4). In our previous clinical study, we found that the subjects defined by 183 mg/dL<1- hour post-challenge plasma glucose (1-h PG) in normal glucose tolerance (NGT) displayed arterial stiffness indicated by higher values of brachialankle pulse wave velocity (baPWV) (5). These findings raised a question that a rapid elevation and subsequent decline of glucose levels can be a regulator for coronary artery endothelial cell proliferation and colon cancer cell proliferation as previously reported (4). To address this issue, acute short term (1 hour) and more chronic long-term (24 hours) effects of glucose on cell proliferation and was compared Erk2 activation in human coronary artery endothelial and human colon cancer cell lines.

This study was approved by the Committee for Safe Handling of Living Modified Organisms in Gunma University and carried out according to the guidelines of the committee. We prepared materials as follows. Phosphor-Erk and α-tubulin antibodies were purchased from Cell signaling. The horseradish peroxidase (HRP) conjugated anti rabbit or mouse immunoglubulin G (IgG) antibody was obtained from Thermo Scientific. Cell culture medium and reagents were from Life Technologies. All of the other chemicals used in this study were purchased from Sigma-Aldrich. Our culture work for current experiments were as follows. Human coronary artery endothelial cells (HCAEC, TAKARA code D10024) were purchased from TAKARA Bio Incorporation (Japan). These cells were maintained in endothelial cell growth medium MV2 at 37˚C with 5% CO2. Human colon cancer cells (HCT116 cells) were purchased from Japanese Collection of Research Bioresources Cell Bank (Japan). These cells were maintained in Dulbecco’s modified Eagle’s medium with 10% fetal bovine serum. The cells were grown to sub-confluence and then incubated with either glucose free culture medium [designated as sample (1)] or culture medium supplied with 183 mg/dL D-glucose [designated sample (2)] or culture medium supplied with 183 mg/dL D-glucose for just one hour (9:00 am to 10:00 am) per day (rest of 23 hours was glucose free condition) [designated as sample (3)] as shown in Figure 1. Two weeks later, cell growth was estimated by the cell proliferation kit II (XTT) assay and the other sets of cells were also washed with cold PBS and stored at -80˚C as frozen dishes at 10:00 am until furtherer western blotting analysis.

Our western blotting procedures are as follows. Details were already described (6, 7). Briefly scraped frozen cells were rocked for 10 minutes at 4˚C with NP-40 lysis buffer (25 mM Hepes, pH=7.4, 10% glycerol, 50 mM sodium fluoride, 10 mM sodium pyrophosphate, 137 mM sodium chloride, 1 mM sodium orthovanadate, 1 mM phenylmethylsulfonyl fluoride (PMSF), 10 μg/mL aprotinin, 1 μg/mL pepstatin, 5 μg/mL leupeptin). Insoluble material was separated from the soluble extract by centrifugation for 10 minutes at 4˚C and the total protein amount in the supernatant was determined by Bicinchoninic acid (BCA) method. Also samples were normalized to total protein content. The samples were resuspended in Sodium dodecyl sulfate SDS sample buffer and heated at 100˚C for 5 minutes. Samples were separated by Sodium dodecyl sulfate polyacrylamide gel electrophoresis (SDS-PAGE) and transferred electrophoretically to polyvinylidene difluoride (PVDF) membranes. The samples were immunoblotted with specific antibody as indicated in figure legends.

**Fig.1:**
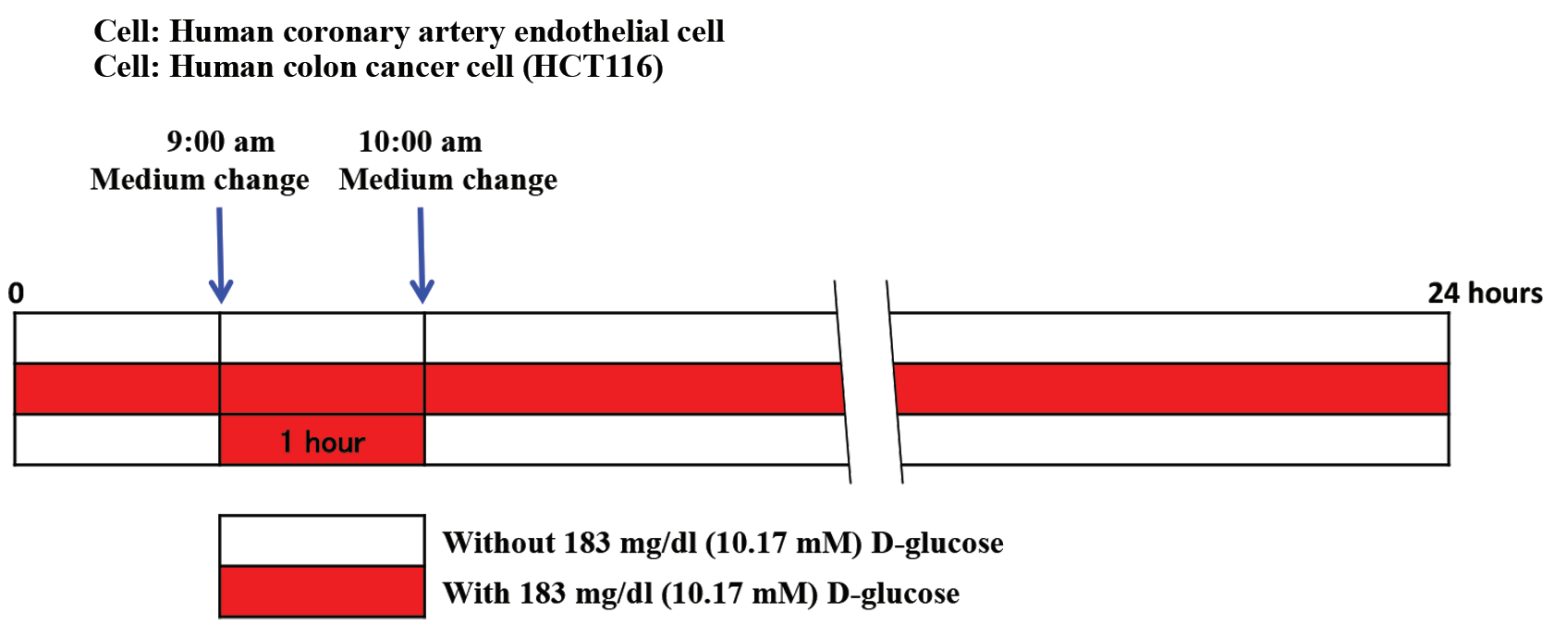
Experimental design. Three different culture conditions are shown. White column represents culture condition without 183 mg/dL D-glucose supplement. Red column represents culture condition with 183 mg/dL D-glucose supplement. The upper column means cells were incubated with culture medium without 183 mg/dL D-glucose supplement. The middle column means cells were incubated with culture medium with 183 mg/dL D-glucose supplement. The bottom column means cells were incubated with 183 mg/dL D-glucose for just one hour per day and during the rest of 23 hours, cells were incubated without 183 mg/dL supplement.

We estimated cell viability by Cell proliferation assay with the cell proliferation kit II (XTT). Details were already described (8). Briefly cell viability was estimated by XTT cell proliferation assay kit following manufacture’s instruction (Roche Applied Science, Japan). Briefly seeded cells were mixed with prepared 2,3-Bis (2-methoxy-4-nitro-5-sulfophenyl)-2H-tetrazolium-5-carbox-anilide (XTT) working solution for 6 hours at 37˚C under 5% CO_2_ and absorbance value obtained at 490 nm with a reference correction at 630 nm.

All data are expressed as mean ± SD in figures. Data were analyzed using 1-factor ANOVA in order to compare the means of all the groups. The Turkey-Kramer multiple comparisons procedure was used to determine statistical differences between the means with a P<0.05 evaluated for statistical significance by InStat 2.00 program.

From those experiments we obtained the following results. First, effect of 183 mg/dL (10.17 mM) glucose addition on coronary artery endothelial cell on proliferation was as follows. Coronary artery endothelial cells were plated at the same density and grown as described in above. Phase contrast microscopy observation revealed that there was a visual increase in the number of cells when incubated with 183 mg/dL D-glucose for 1 hour per day and without 183 mg/dL supplement during the rest of 23-hours compared to cells maintained in the absence or presence of 183 mg/dL D-glucose supplement (Fig.2A). Quantification of cell proliferation performed by XTT analyses demonstrated a statistically significant increase in proliferation when the cells were treated with 183 mg/dL D-glucose for 1 hour per day and without 183 mg/dL supplement during the rest of 23 hours compared to cells maintained in the absence or presence of 183 mg/dL D-glucose supplement (Fig.2B). Second, the effect of 183 mg/dL (10.17 mM) glucose on phospho-Erk western blotting of coronary artery endothelial cell proliferation was as follows. Western blotting analysis of the cell extracts for pT202/Y204- Erk indicated increase of Erk2 phosphorylation when the cells were grown with 183 mg/dL D-glucose for 1 hour per day and without 183 mg/dL supplement during the rest of 23 hours compared to cells grown in the absence of 183 mg/dL D-glucose supplement (Fig.3A). Surprisingly, when the cells were grown with 183 mg/dL D-glucose, Erk2 phosphorylation was significantly decreased compared to cells grown in the absence of 183 mg/dL D-glucose supplement (Fig.3A, upper panel). Quantification results are shown in Figure 3B. α-tubulin blotting suggested that equal amount of protein was loaded in each lane (Fig.3A, lower panel).

Third, effect of 183 mg/dL (10.17 mM) glucose addition on colon cancer cell proliferation was as follows. Human colon cancer cells (HCT116 cells) were plated at the same density and grown as described in the materials and methods section. Phase contrast microscopy observation revealed that there was a visual increase in the number of cells when incubated with 183 mg/ dL D-glucose compared to cells with either the absence of 183 mg/dL D-glucose supplement or 183 mg/dL D-glucose supplement for 1 hour per day and without 183 mg/dL supplement during the rest of 23 hours (Fig.4A). Quantification of cell growth performed by XTT analyses demonstrated a statistically significant increase in proliferation when the cells were treated with 183 mg/dL D-glucose in comparison with cells maintained either in the absence of 183 mg/dL D-glucose supplement or 183 mg/ dL D-glucose for 1 hour per day and without 183 mg/dL supplement during the rest of 23 hours (Fig.4B). Fourthly, effect of 183 mg/ dL (10.17 mM) glucose on phospho-Erk western blotting of human colon cancer cell proliferation was as follows. Western blotting analysis of cell extracts for pT202/Y204-Erk indicated increased Erk2 phosphorylation when the cells were grown with 183 mg/dL D-glucose compared to the cells either in the absence 183 mg/dL D-glucose or with 183 mg/dL D-glucose for 1 hour per day supplement and without 183 mg/dL supplement during the rest of 23 hours (Fig.5A upper panel). Quantification results are shown in Figure 5B. α-tubulin blotting suggested that equal amount of protein was loaded in each lane (Fig.5A, lower panel).

In this paper we examined the effect of high level of glucose on coronary artery endothelial and human colon cancer cell proliferation. We found that rapid glucose elevation for 1 hour followed by glucose depletion for the next 23 hours increased coronary artery endothelial cell growth compared to cells maintained in the absence or presence of 183 mg/dL D-glucose. On the other hand, colon cancer cell proliferation was not affected by the acute increase in glucose levels. These results were supported by the results of phosphor-Erk2 western blotting analysis. However, human colon cancer cell proliferation was increased under 183 mg/dL D-glucose supplemented condition and those results were not observed in the case of coronary artery endothelial cells. In addition to these results, it is necessary to study about other signal pathway such as phosphoinositide 3-kinase (PI3-K)-Akt signal pathway as in the previous studies, high glucose exposure promote colon cancer cell proliferation as PI3-K dependent manner (4) and PI3-K is activated by high glucose exposure in human omental arterial cell (9).

**Fig.2:**
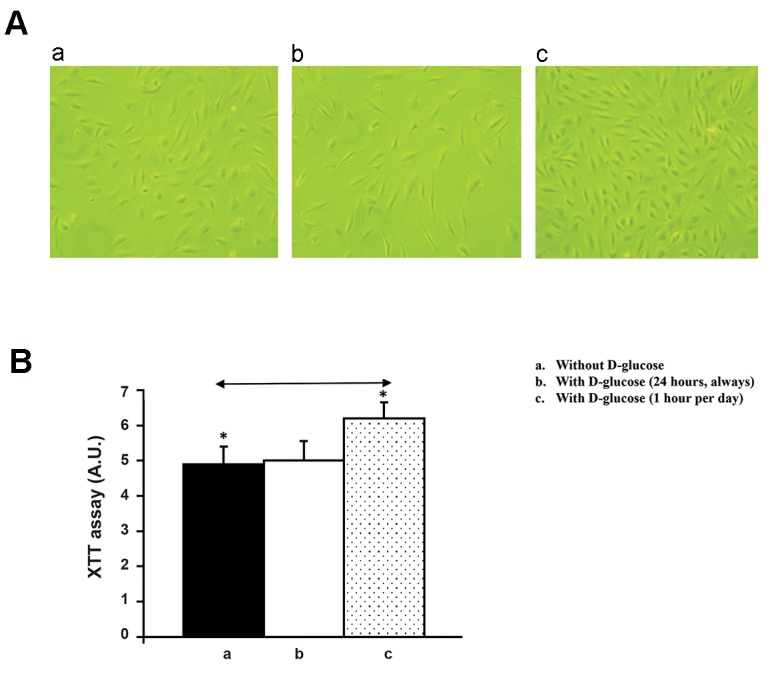
Effect of 183 mg/dL (10.17 mM) D-glucose on coronary artery endothelial cell proliferation. A. Phase contrast microscopic observation results are shown. Coronary artery endothelial cells were grown in without 183 mg/dL D-glucose supplement (a) or with 183 mg/dL D-glucose supplement (b) or with 183 mg/dL D-glucose for just one hour per day and during the rest of 23 hours, cells were incubated without 183 mg/dL supplement (c). These are representative fields independently performed five times and B. The results of phase contrast microscopic observation are quantitated by XTT assay as described in methods section. Closed column represents without 183 mg/dL D-glucose supplement. Open column represents with 183 mg/dL D-glucose. Dotted column represents with 183 mg/dL D-glucose for just one hour per day and during the rest of 23 hours, cells were incubated without 183 mg/dL supplement. *; P<0.05.

**Fig.3:**
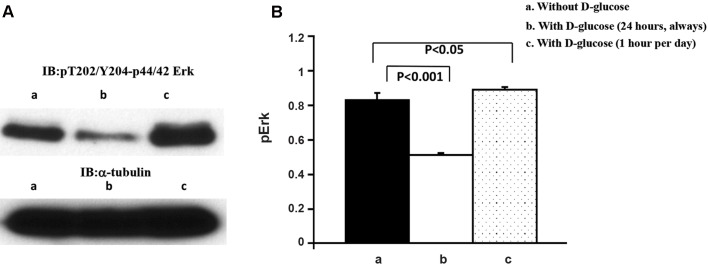
Effect of 183 mg/dL D-glucose on phosphor-Erk western blotting of coronary artery endothelial cell proliferation. A. Coronary artery endothelial cells were grown under the three different culture conditions as described in methods section and represented in Figure 1. Cell extracts were prepared and western blotting for Erk phosphorylation (threonine 202 and tyrosine 204) was performed. Loaded protein amount was estimated by α-tubulin western blotting and B. Erk phosphorylation grade was normalized by α-tubulin levels and results are shown in the bar graphs. Closed column represents without 183 mg/dL D-glucose supplement (a). Open column represents with 183 mg/dL D-glucose supplement (b). Dotted column represents with 183 mg/dL D-glucose for just one hour per day and during the rest of 23-hours, cells were incubated without 183 mg/dL supplement (c). These are representative western blotting independently performed four times.

**Fig.4:**
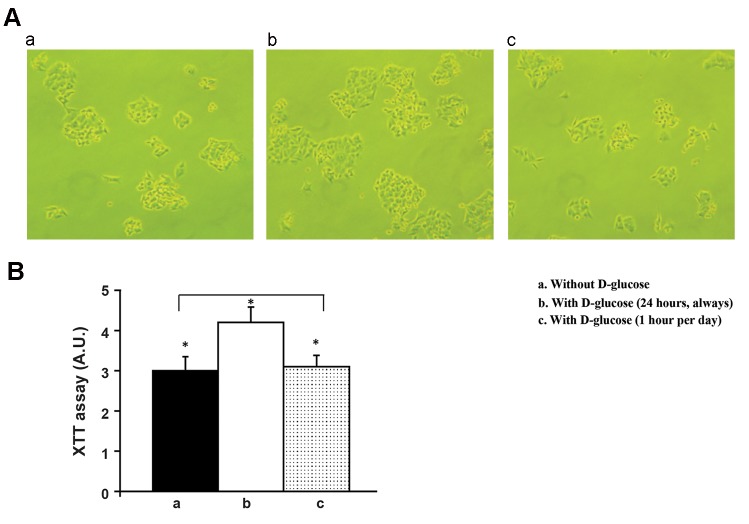
Effect of 183 mg/dl (10.17 mM) D-glucose on colon cancer cell proliferation. A. Phase contrast microscopic observation results are shown. HCT116 human colon cancer cells were grown in either without 183 mg/dL D-glucose supplement (a) or with 183 mg/dL D-glucose (b) or with 183 mg/dL D-glucose for just one hour per day and during the rest of 23-hours, cells were incubated without 183 mg/dL supplement (c). These are representative fields independently performed four times and B. Phase contrast microscopic observation results are quantitated by XTT assay as described in methods section. Closed column represents without 183 mg/dL D-glucose supplement (a). Open column represents with 183 mg/dL D-glucose (b). Dotted column represents with 183 mg/dL D-glucose for just one hour per day and during the rest of 23-hours, cells were incubated without 183 mg/dL supplement (c). *; P<0.05.

**Fig.5:**
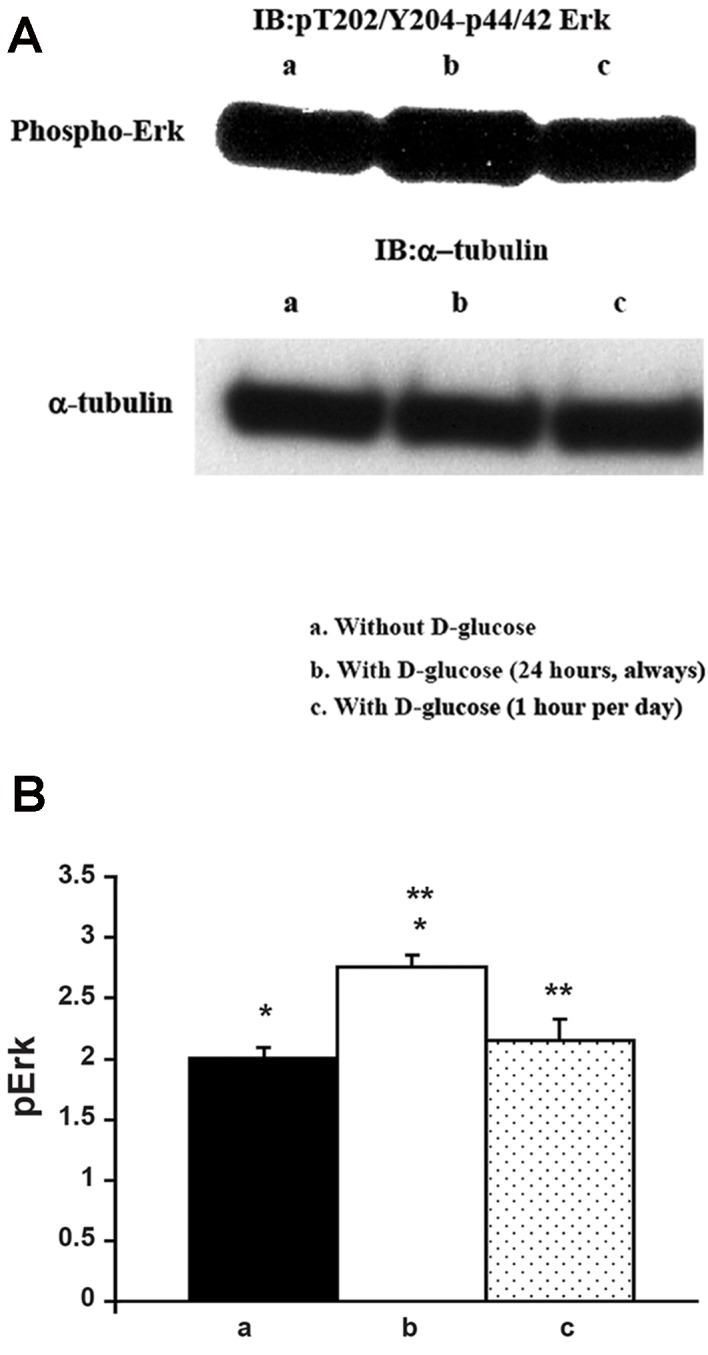
Effect of 183 mg/dl (10.17 mM) D-glucose on phospho-Erk western blotting of colon cancer ell proliferation. A. HCT116 human colon cancer cells were grown under the three different culture conditions as described in methods section and represented in Figure 1. Cell extracts were prepared and western blotting for Erk phosphorylation (threonine 202 and tyrosine 204) was performed. Loaded protein amount was estimated by α-tubulin western blotting. These are representative western blotting independently performed four times and B. Erk phosphorylation grade was normalized by α-tubulin levels and results are shown in the bar graphs in Figure 3B. Closed column represents without 183 mg/dL D-glucose supplement (a). Open column represents with 183 mg/dL D-glucose supplement (b). Dotted column represents with 183 mg/dL D-glucose for just one hour per day and during the rest of 23-hours, cells were incubated without 183 mg/dL supplement (c). *; P<0.001 and **; P<0.01.

Our results may also account for why individuals with impaired glucose tolerance (IGT) as well as those with diabetes mellitus have increased risk of atherosclerosis. On the other hand, in terms of high level of glucose exposure effect on human colon cancer cells, our results were different that previously reported (3). This discrepancy may be due to different glucose level studied. Yasunari etc used glucose at approximately 400 mg/dL in comparison to 183 mg/dL glucose used in our study. We selected 183 mg/dL as this more closely represents the glucose excursions *in vivo*, as it is very rare that blood glucose levels of diabetes mellitus patients are maintained around 400 mg/dL levels.

Tumor cells cover energy requirements by anaerobic glycolysis and have a high demand on glucose. At the same time, tumor cells must often survive in a hostile tumor microenvironment with decreased availability of nutrients. One strategy utilized by some tumor cells is the up-regulation of membrane transporters such as the high affinity sodium/glucose cotransporter-2 (SGLT-2) that may be the primary transporter responsible for glucose take-up (10). We reported that human colon cancer cells (HCT116 cell) express SGLT-2 (8) and these results might explain that rapid glucose elevation/depletion causes coronary artery endothelial cell proliferation but sustain high level of glucose stimulates human colon cancer cell proliferation.

Taken together, these data suggest that limiting elevated glucose excursions may be an important strategy to inhibit the development of atherosclerosis and colon cancer progression. Further studies are necessary to expand these cultured cell findings *in vivo* to examine particularly the effects of glucose on animal models and human cancer patients.
